# Evolution, revolution and heresy in the genetics of infectious disease susceptibility

**DOI:** 10.1098/rstb.2011.0275

**Published:** 2012-03-19

**Authors:** Adrian V. S. Hill

**Affiliations:** Wellcome Trust Centre for Human Genetics, University of Oxford, Oxford OX3 7BN, UK

**Keywords:** susceptibility, genomics, pathogen-driven selection, evolution, diversity, exome

## Abstract

Infectious pathogens have long been recognized as potentially powerful agents impacting on the evolution of human genetic diversity. Analysis of large-scale case–control studies provides one of the most direct means of identifying human genetic variants that currently impact on susceptibility to particular infectious diseases. For over 50 years candidate gene studies have been used to identify loci for many major causes of human infectious mortality, including malaria, tuberculosis, human immunodeficiency virus/acquired immunodeficiency syndrome, bacterial pneumonia and hepatitis. But with the advent of genome-wide approaches, many new loci have been identified in diverse populations. Genome-wide linkage studies identified a few loci, but genome-wide association studies are proving more successful, and both exome and whole-genome sequencing now offer a revolutionary increase in power. Opinions differ on the extent to which the genetic component to common disease susceptibility is encoded by multiple high frequency or rare variants, and the heretical view that most infectious diseases might even be monogenic has been advocated recently. Review of findings to date suggests that the genetic architecture of infectious disease susceptibility may be importantly different from that of non-infectious diseases, and it is suggested that natural selection may be the driving force underlying this difference.

## 1. Why study the genetics of infectious disease susceptibility?

Studies of genetic susceptibility to infectious diseases in humans have a long history, starting with blood groups and other phenotypic markers and a striking success in the early 1950s with the identification of the sickle haemoglobin variant as a major resistance factor for malaria [1]. Today, a great variety of approaches are used from twin studies to whole-genome sequencing. These efforts have several goals.

Foremost is a better understanding of disease pathogenesis and resistance in the expectation that this will lead, in time, to improved interventions such as better drugs or vaccines to prevent or attenuate the great global burden of infectious disease morbidity and mortality. With over 10 million deaths annually from infectious diseases and the threat of new epidemics and pandemics, this is a very high priority. There are clear examples where human genetic research has supported the development of new drugs and vaccines. A recently introduced class of human immunodeficiency virus (HIV) drugs known as entry inhibitors were developed in part based on the knowledge that a specific deletion in the gene for the CCR5 co-receptor greatly reduced the risk of HIV infection and the rate of disease progression after infection [2]. The leading vaccine candidate for *Plasmodium vivax* malaria [3], the Duffy binding protein, was identified and developed supported by evidence that genetic absence of its red blood cell entry receptor provides almost complete protection from this type of malaria [4]. Awareness of the prevalence of a common immunodeficiency of mannose-binding lectin, conferring increased risk of bacterial disease, has encouraged the development of replacement therapy [5].

A second application that is gaining increasing attention is the potential to stratify populations for risk of infectious disease based on genetic profiling. This has not been a priority until now as most preventive interventions such as childhood vaccines have been aimed at universal coverage. However, as more potentially useful vaccines are licensed and the costs of new vaccines escalate, targeted use is becoming a consideration. When a genetic profile costs less than a vaccine and the profile has many other applications in predicting disease risk, it may well be cost-effective to target newer vaccines to those who will benefit most from them. The recent awareness that low-frequency large-effect variants may make a large contribution to inter-individual genetic variation in susceptibility to many diseases [6] should increase interest in defining, early in life, the constellation of potentially deleterious variants that comprise an individual's inheritance.

But the third, and one of the most interesting aspects of this field, is that most relevant to the theme of this issue. The evolutionary significance of genetic variation in susceptibility to infection has long fascinated the public as well as physicians and infection specialists. Questions such as the importance of infectious diseases in generating and maintaining the great diversity that we can now readily define in our genomes have long been debated. I will discuss some aspects of evolutionary interest towards the end of this review, after first providing an overview of approaches and recent progress in this field. The focus will be on information that has been provided by large well-designed case–control studies, which have provided the most compelling evidence of the relevance of specific genetic variants to infectious disease susceptibility.

## 2. Infectious disease susceptibility is genetically controlled

There are some well-studied examples of familial clustering of severe infectious disease syndromes, and these very rare monogenic disorders have been reviewed elsewhere [7]. A more challenging question is the extent to which common major infectious diseases are affected by host genetics. Here, the standard genetic measure used for complex traits, lambda-s, a measure of the increase in risk to siblings of an affected case compared with an unrelated individual, is confounded by the tendency of people to live with their relatives, so that dissecting the effect of environment from shared genes become very difficult. A better approach is to compare the concordance of disease in fraternal and identical twin pairs, where a greater concordance in the latter provides a measure of heritability. Such studies have been undertaken for several infectious diseases, usually many years ago, and a relatively consistent picture of significant heritability for chronic infectious diseases emerges. The evidence is less clear for acute infections such as measles, where exposure and infection rates were very high when these early twin studies were performed [8]. But in tuberculosis [9], leprosy [10], *Helicobacter pylori* infection [11], chronic hepatitis B infection [12] as well as in the phenotype of immune responses to vaccination [13,14], there is evidence of greater concordance in monozygotic compared with dizygotic twin pairs.

## 3. Approaches to gene identification

One of the most interesting aspects of this field is the wide variety of approaches that can be, and have been, taken towards identification of relevant susceptibility genes for infectious diseases.

Some have advocated the use of animal models of infectious diseases as a route to finding human infectious diseases susceptibility loci [15]. It is clear that rodent strains can differ strikingly in susceptibility to infections and genes can be mapped and identified in controlled challenge studies. The difficulty has been in extrapolating these findings to humans who will often not show comparable polymorphism in the relevant loci or may even lack a homologous gene. A related approach is to define the susceptibility profile of artificially generated knockout mice. Such efforts continue and are clearly of value in identifying pathways of potential relevance to human infectious disease susceptibility, but in terms of actual definition of known human susceptibility loci, very few of these have emerged from animal model analysis. Recently, a tuberculosis susceptibility gene was suggested by an analysis of zebrafish genetics [16], but the homologous gene was not associated with human tuberculosis susceptibility in a very large study of over 3700 clinical cases [17].

Prior to the availability of genome-wide association technologies, linkage mapping was attempted to map major genes in common infectious diseases. This required the laborious collection of families with multiple cases, usually affected sibling pairs, and the use of microsatellite markers to define chromosomal segments segregating with disease. It was possible to identify a small number of regions linked to leprosy [18–20], tuberculosis [21,22] and chronic hepatitis [23] in these family studies and some positional candidate genes were associated with disease. But it has been difficult to define the mapped loci in large-scale association studies and to date none appears to have been independently confirmed. These searches for genetic loci of very large effect that might be detectable in such small-scale linkage studies were encouraged by a form of pedigree analysis known as complex segregation analysis, which was claimed to identify the existence (but not the location) of major loci for many infectious diseases [24,25]. However, the clearest result from these genome-wide linkage studies is that such prevalent major genes must be very rare in infectious diseases, in contrast to some autoimmune diseases where major loci, especially in the human leucocyte antigen (HLA) complex, could be detected by affected sibling pair studies. In summary, the central problem with this approach is that most infectious disease susceptibility genes have too small effect sizes and/or too low a frequency to allow detection in linkage studies of even hundreds of families.

After this foray into family studies, the field returned to association studies of infectious disease susceptibility comparing the frequency of variants in affected cases with those of healthy controls. Ever since the successful identification of sickle haemoglobin as a resistance locus in the 1950s [1], and extensive blood group association studies in the 1960s [26], such case–control studies have been the most widely used and most successful approach to identifying convincing infectious disease susceptibility genes in humans.

However, the very simplicity of the approach and the reducing cost of genotyping have generated their own problems. A proliferation of studies of modest size, and with apparently statistically significant results, led to a greater focus of thresholds for claiming statistical significance and to a preference, and later a requirement, for replication studies to ‘confirm’ an association. More recently, with the advent of whole-genome association arrays and very recent exome and whole-genome sequencing studies, criteria for ‘statistical significance’ are being assessed again. We remain in a transitional stage where some journals will accept reports with significant levels as modest as *p* < 0.05, and others will require replication studies and a ‘genome-wide significance’ level of *p* < 5 × 10^−7^ or less. This dramatic difference in publication thresholds complicates review of the field as well as interpretation of results, as does the long-standing [27] tendency for positive results to be published more readily than negative ones.

## 4. Candidate gene studies

I shall start with some examples of infectious disease associations that appear convincing and are unusual, in that the effect size is very large. All of these are effectively candidate gene studies, in that the investigators chose to assess these particular genes based on some biological information rather than a genome-wide scan. I am aware of six associations that are major effects [28], arbitrarily defined as an odds ratio exceeding 3, where the variant allele is polymorphic in some human populations. These six (table 1) are all supported by case–control analysis of allele and genotype frequencies but, interestingly, three were discovered in a surprising manner. The first reports of the sickle haemoglobin [1] and Duffy blood group associations [4] with malaria resistance included analysis in deliberately infected volunteers, as did the identification of the importance of the non-secretor status for blood group antigens, determined by the fucosyltransferase (*FUT2*) gene, in resistance to infection by norovirus [31], a frequent viral cause of diarrhoeal disease. The ability to identify these associations in small microbial challenge studies reflects both the large effect sizes of these variants and excellent biological insights suggesting the relevant candidate gene.

**Table 1.** ‘The Big Six’ of human infectious disease genetics. All six genetic variants have a substantial impact (odds ratio > 3) on risk of an important infectious disease and all are prevalent in at least one major human population group.

genetic variant/condition	gene	infectious disease	year reported	reference
sickle haemoglobin	*HBB*	*Plasmodium falciparum* malaria	1954	[1]
the Duffy blood group	*DARC*	*Plasmodium vivax* malaria	1976	[4]
prion protein gene variant	*PRNP*	Creutzfeldt–Jakob disease	1991	[29]
Melanesian ovalocytosis	*SLC4A1*	*P. falciparum* malaria	1995	[30]
CC chemokine receptor-5 *Δ*32	*CCR5*	HIV-1 infection	1996	[2]
blood group non-secretion	*FUT2*	Norwalk virus diarrhoea	2003	[31]

In contrast, other candidate gene associations (table 2) that appear compelling typically have smaller effect sizes, with odds ratios of 0.5–2, but most of these are still larger effects than are generally found by genome-wide analyses of non-infectious diseases (with the sole exception of HLA associations with autoimmunity where occasional exceptionally strong associations are seen).

**Table 2.** Loci strongly associated with infectious disease susceptibility. For each gene–disease pair the table indicates whether the association was identified by, or confirmed by, a genome-wide association study (GWAS), whether the association should be detectable by exome sequencing studies and whether the minor allele is associated with protection or susceptibility (or whether different alleles are associated with protection and susceptibility = ‘both’).

gene	disease	GWAS?	exomic?	minor allele	reference
haemoglobin S	malaria		yes	protective	[1]
*SLC4A1* (ovalocytosis)	malaria		yes	protective	[30]
*CCR5*	HIV/AIDS		yes	protective	[2]
*PRPN*	vCJD		yes	protective	[29]
*FUT2*	norovirus		yes	protective	[31]
Duffy blood group	vivax malaria		no	protective	[4]
*HLA-DR/DQ*	leprosy	confirmatory	yes	both	[32]
*HLA-B*	HIV/AIDS	confirmatory	yes	both	[33]
*HLA-DQ/DP*	HBV	yes	yes	both	[34]
*HLA-C*	HIV/AIDS	confirmatory	yes	both	[33]
blood group O	malaria		yes	protective	[35]
*G6PD*	malaria		yes	protective	[36]
*CFH*	meningococcus	confirmatory	yes	protective	[37]
*MAL*	bacteraemia		yes	protective	[38]
*TLR1*	leprosy	confirmatory	yes	protective	[39]
*IL-28B*	HCV	yes	yes	susceptible	[40]
*MBL2*	pneumococcus		yes	susceptible	[41]
*C13orf31*	leprosy	yes		susceptible	[32]
*CCDC122*	leprosy	yes		protective	[32]

It is impossible to review in any detail here the plethora of reports of candidate genes that have now been associated with various infectious diseases, and there have been several recent reviews [28,42] and an entire monograph on this subject [43]. Some of the more compelling associations are listed in table 2, with references. Instead, I will discuss some selected candidates of immunological relevance that may impact on the risk of disease from several infectious pathogens. Associations with susceptibility to several infectious diseases may of course increase the evolutionary importance of specific genetic variants.

## 5. Innate immune signalling pathways

An explosion of research in innate immunity over the last decade has led to considerable clarification of the role of several classes of innate receptors in triggering inflammation and acquired immunity [44]. The toll-like receptor (TLR) signalling pathway has been the best characterized leading to a particular emphasis on polymorphisms in this pathway by genetic studies. A striking example is the prevalence of a variant of one of the four TLR signalling adaptors, named MAL (encoded by the gene *TIRAP*) in many populations [38]. This appears to be a functional knockout allele, and heterozygotes have been reported to have an approximately twofold reduction in risk of several infectious diseases, including pneumococcal disease, tuberculosis and malaria [38]. The homozygous genotype is rare in many populations but in Europe, where this genotype is less uncommon, homozygotes may be at a slightly increased risk of bacterial disease [38]. If so, this surprising genotypic pattern could contribute to the evolutionary maintenance of this polymorphism over long periods of time.

Another example of an immunological gene that impacts on multiple infectious diseases was reported recently. Cytokine-inducible SRC homology 2 domain protein (CISH) was the first member of the regulatory suppressor of cytokine signalling (or SOCS) family to be described, and *CISH* is the gene most consistently upregulated by interleukin (IL)-2 stimulation in humans, and is a key negative regulator of cytokines, especially IL-2. Single-nucleotide polymorphisms (SNPs) flanking the promoter of this gene were associated with risk of bacteraemia, tuberculosis and severe malaria in diverse populations and at least one of these SNPs was shown to be functionally relevant [45].

A coding change at position 602 in the TLR1 protein, a key innate receptor component for many bacteria, was shown to impact on the ability of this receptor to reach the cell surface. The variant is found at low frequencies in Africa, but is the more frequent allele in many Caucasian populations. This variant has recently been associated with a substantial reduction in risk of leprosy in Turkey and India, with the cumulative data reaching impressive statistical significance (*p* < 10^−7^) [39,46]. Intriguingly, the same variant was associated with a reduced risk of tuberculosis in an exon resequencing study of TLR genes in African Americans [47]. So this may provide another example of a variant impacting on several diseases, and in this case, there is suggestive evidence that the protective variant may have increased in frequency by providing protection against mycobacterial disease.

Finally, one of the best studied genes impacting on a wide variety of autoimmune diseases is the lymphoid tyrosine phosphatase (Lyp) gene, *PTPN22*. An R620W change is common in Europeans and increases the risk of type I diabetes, rheumatoid arthritis, systemic lupus erythematosus and several other autoimmune diseases [48]. Interestingly, the same variant was associated with an increased risk of invasive pneumococcal disease and empyema (a form of suppurative lung disease) [49] and very recently with leprosy in India [50]. Although it might be expected that alleles associated with an increased risk of autoimmunity might protect against infectious diseases, in this case the observed association is in the opposite direction.

Many other infectious disease associations with genetically variable components of the innate and acquired immune systems are appearing. Several, such as the relevance of variants in genes encoding inhibitors for NFκB signalling [51–53], have been studied only in a small number of infections (in this case pneumococcal disease), and more extensive surveys of infectious diseases are justified. The challenge continues to be accessing large well characterized clinical series with suitable controls to allow adequately powered studies.

In summary, just as HLA genes may impact on risk of several infectious diseases as well as autoimmunity, there are now several examples of non-HLA loci that also appear relevant to multiple infections as well as examples of rare monogenic variants that impact on the risk of several infectious diseases [54]. The consequences of this for evolutionary change in allele frequencies over time will undoubtedly be complex. Not only will selection pressures fluctuate with the prevalence and strain of infectious pathogens (see below), but the presence of different pathogens might have either opposing or additive impacts on the change in allele frequencies of particular genetic variants.

## 6. Genome-wide association studies

The introduction of genome-wide association studies (GWAS) has revolutionized the field of complex disease genetics. The availability of millions of SNPs mapped across the human genome and of microarrays that allow cost-effective genotyping of millions of SNPs in thousands of individuals provided, for the first time, the opportunity to test fairly comprehensively for genetic markers that would tag causative variants [55]. In many diseases, the yield of this approach has been spectacular, with hundreds of loci now identified in, for example, autoimmune diseases. This is providing new insights into the molecular pathogenesis of these diseases as a route to designing and developing new treatments. There is also potential value in devising diagnostic arrays that would allow some prediction of risk of certain diseases.

Despite these spectacular successes, there are limitations to this approach. Even in the diseases where many dozens of loci have been identified and replicated, the proportion of the estimated genetic variance that can be explained by all the identified genetic associations is modest, often less that 20 per cent of the total genetic variances suggested by twin studies [56]. This is because the individual associations are generally of modest magnitude, with odds ratios typically of 1.1 to 1.5. Furthermore, there is little evidence of epistatic interactions between loci that could account for some of the missing genetic component. Searches for structural variants that might account for the missing component have met with little success. The leading current hypothesis to explain the missing component is that rare variants, perhaps with larger effect sizes, are cumulatively responsible [56], but because of their low frequencies these are very poorly assessed with available GWAS microarrays.

**Figure 1.**
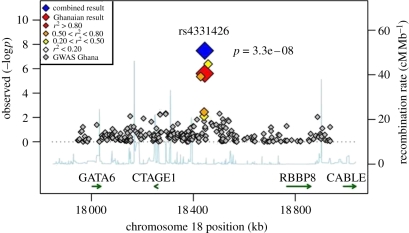
Association plot of the main associated locus identified in combined analysis of genome-wide association studies of tuberculosis in The Gambia and Ghana. The *y*-axis show the negative log of the *p*-value for the association test. The peak of association is in a gene-poor region, but the positions of flanking genes are shown. Adapted from Thye *et al*. [57].

For infectious diseases, the picture is yet more complicated. Fewer diseases have been studies by GWAS, in part because the approach requires very large sample sizes to be worthwhile. This results from the very large number of SNPs assayed, leading to a major challenge in sorting true signals from the noise or false positives that inevitably turn up when assaying about a million variants. The general approach is to require replication of positive signals and to use a challenging threshold level of ‘genome-wide’ statistical significance of about *p* = 5 × 10^−7^. This requirement for typically thousands of cases and suitable matched controls has limited GWAS studies of infectious diseases. Despite the high prevalence of many infectious diseases in developing countries, resource limitations still make it harder to collect 2000 tuberculosis cases in West Africa than 20 000 diabetes cases in western Europe. A further challenge is found in studies of African populations, which were among the first to be studied by GWAS for infectious diseases, as part of the Wellcome Trust Case–Control Consortium [57,58]. The shorter extent of linkage disequilibrium in Africans compared with non-Africans leads to a requirement for larger numbers of SNPs to attain the same coverage of the genome as in Caucasian and Asian populations. Greater diversity in populations in Africa and complexities in haplotype structure may also complicate fine mapping of causative SNPs (figure 1) [58].

Nonetheless, several GWAS studies of infectious diseases have been undertaken with some notable successes. In Africa, a combined analysis of tuberculosis cases from The Gambia and Ghana identified a signal with genome-wide significance on chromosome 18 [57]. Although this region is gene-poor, a flanking gene that encodes the transcription factor GATA6 is an excellent positional candidate for tuberculosis. Another signal was found on chromosome 2 in the *PARD3B* cell polarity gene [57], which interestingly also emerged from a GWAS of HIV disease progression [59] (although the associated regions of the gene in the two studies are different). Further statistical analysis of the tuberculosis GWAS datasets using an imputation technique to estimate association with less well-covered genomic regions has identified a further highly significant signal [60]. A parallel GWAS study of malaria in Gambians identified the haemoglobin AS variant with clear statistical significance but highlighted the challenges of using limited microarray coverage to map even such strong hits in Africans [58]. A very striking positive association was reported for hepatitis C where one of the interferon lambda genes, *IL-28B*, showed a striking association with viral clearance and with response to treatment [40].

But probably the most marked GWAS success in infectious diseases has been a study of leprosy reported by a Chinese group [32]. By studying about 4000 cases and 7000 controls, five new genes showed strongly significant associations (*p* < 10^−10^) in addition to the expected HLA class II region association. Three of these six genes could be replicated in an independent study of Indian patients with leprosy [61]. Most interestingly, the susceptibility genes identified in Chinese leprosy show a striking overlap with genes previously associated with Crohn's disease [32]. Although this does not necessarily implicate mycobacteria in the aetiology of Crohn's disease, a theory for which there is some support [62], this does point to the same pathways, related to NOD2 signalling, being relevant in these two granulomatous diseases.

In contrast, large GWAS efforts in HIV/AIDS have been less successful [63]. Although the associations in the HLA class I regions with rate of progression to AIDS were reconfirmed and well mapped, no convincing new associations were identified in other chromosomal regions [33,64]. However, these GWASs of disease progression have been limited to non-African populations and studies in the continent most affected by the epidemic and with the greatest human genetic diversity should still be worthwhile. Similarly, a somewhat smaller GWAS in meningococcal disease confirmed an association with the complement factor H (*CFH*) gene, previously identified in a candidate gene study of this disease [37]. And in malaria a first GWAS in Gambians failed to identify new loci with genome-wide significance [58], although large-scale GWASs in other African populations are continuing as part of the extensive MalariaGEN consortium [65].

## 7. Genetic architecture of infectious diseases

The limited success, at least to date, of GWAS approaches in infectious diseases raises the question of whether this is the most efficient means of identifying the genetic factors underlying variable susceptibility. Indeed, for many non-infectious diseases the field is moving towards next-generation sequencing approaches to try to identify the genes accounting for the heritability not identified through GWAS. This discussion raises the key question of the genetic architecture of variable susceptibility to disease (table 3). There are at least three competing views. GWAS analysis was based on the hypothesis that most of the genetic component to common diseases can be explained by common variants; this is sometimes dubbed the common disease–common variant hypothesis [66]. Because GWAS relies on linkage disequilibrium between common genotyped markers and relatively common causative variants, it has inadequate power to detect significant associations with rare causative variants. The second hypothesis listed in table 3 is that rare monogenic variants with high penetrance account for the bulk, or all, of the genetic component to common disease [67]. Although this may sound implausible, given the lack of Mendelian inheritance of most common disease, it has been seriously proposed for at least paediatric infectious diseases [68]. This idea emerged from the primary immunodeficiency disease field where it has been fairly straightforward to identify some causal mutations by sequencing of candidate genes in rare pedigrees with familial severe susceptibility or rare novel mutations. Emboldened by success with these rare phenotypes, one group has suggested that the same monogenic major gene genetic architecture underlies common infectious disease phenotypes [68,69].

**Table 3.** Theories of the genetic architecture of human infectious disease susceptibility. Theory 1 suggests that most relevant genetic variation is encoded by relatively common variants that cumulatively account for most of the genetic variance. Theory 2, in complete contrast, is the extreme view that most relevant genetic variation is encoded by very rare new mutations that have almost complete penetrance, as in primary immunodeficiency diseases. The more recent theory 3 suggests a predominant role for many individually rare variants with incomplete penetrance, which are generally not new mutations, but cumulatively account for most of the genetic component to infectious disease susceptibility.

genetic architecture of infectious diseases susceptibility: three theories	
1	*common variants*
	higher frequency variants
	identifiable by genome-wide association scans
2	*very rare monogenic variants*
	highly penetrant mutations
	mainly novel mutations
3	*multiple rare diverse variants*
	not fully penetrant
	mainly standing genetic variation

A newer and more plausible third hypothesis (table 3) is advocated here for infectious diseases. This is the concept that much of the genetic component to common infectious disease may be accounted for by the cumulative effects of many rare mutations with limited penetrance [66]. While, in this view, rare mutations would account for much of the genetic component, a consensus view would allow for hypothesis 1 to also be partly true, with common variants accounting for part of the genetic component: of course, there is good evidence that this is the case for many diseases (as reviewed earlier). Finally, one should allow that rare familial cases of severe susceptibility, usually in young children, can indeed be Mendelian as has been documented in selected rare cases [67]. However, the lack of evident Mendelian transmission in most cases of infectious disease susceptibility and the evolutionary consideration that severe deleterious mutations should be rapidly eliminated from populations indicates that the contribution of highly penetrant deleterious mutations to the great majority of infectious disease cases must be very limited.

There is a practical relevance to the understanding of which genetic architecture is most correct. If common variants are of over-riding importance, an extension of the GWAS approach, with microarrays of improved coverage and analysis of larger sample sizes, might be the best way forward. But if rare variants account for most susceptibility, sequencing approaches will be required. Fortunately, next-generation sequencing costs continue to fall rapidly and promise to allow hypotheses 2 and 3 to be assessed in the foreseeable future.

## 8. Towards exomics

Sequencing costs remain a major limitation today and, even though whole genome sequencing of over three trillion base pairs is feasible, the costs of full genomic sequencing of thousands of samples in just one infectious disease are still prohibitive. Fortunately, sequencing of just the exome, composed of all the exons in the genome, and some limited exon flanking sequence, might provide a cost-effective alternative [70–72]. This is feasible using a variety of capture matrices. The exome comprises just one per cent of the human genome, and costs per sample are falling rapidly from thousands to hundreds of pounds per sample. Although costs will still be high initially, a limited number of samples can be sequenced for the full exome, to search for suggestive associations, and these can be reassessed in a replication study by more limited sequencing of genes of interest on larger sample numbers. As well as analysing individual mutations, the mutational load in a gene, comprising the full set of coding changes identified, can be compared between cases and controls.

Initial data on exon sequencing in infectious diseases are encouraging. Smirnova *et al*. [73] focused on the *TLR4* gene in meningococcal disease and identified a substantial excess of rare coding changes in cases compared with those in controls, with a remarkably large suggested effect size. Ma *et al*. [47] studied five TLR genes and again found an excess of rare (and some more frequent) coding changes in tuberculosis cases compared with controls.

An argument against focusing on exons is that when one reviews the variants that have been associated with non-infectious disease susceptibility using GWAS analyses, there is strong evidence that the majority of changes identified are regulatory or at least non-coding changes [55]. If this is also true of infectious diseases, then exome sequencing could miss most of the relevant changes. However, I suggest that matters may be different for infectious diseases, based on review of the available data on known associations. Table 2 lists the majority of the most convincing associations with infectious diseases where the causative genetic changes are both polymorphic in human populations and reasonably well defined. It is striking that in sixteen out of seventeen cases, the association could have been identified by exome sequencing. Either the mutation is in an exon or, as in the case of the *IL-28B*–hepatitis C association, a change in an exon tracks very well a flanking (possibly regulatory) change. This excess of exonic changes contrasts strikingly with GWAS-identified changes in say autoimmune diseases. A second difference is also evident. In most GWAS studies of non-infectious diseases, the minor allele is associated with an increased risk of disease whereas, as indicated in table 2, there are more examples of protective than susceptible minor alleles in infectious disease associations.

There are many possible reasons for these striking differences, including some differences in ascertainment methods, but if these differences are real a likely contributor is the action of natural selection by infectious pathogens. In well-studied examples, such as malarial selection of haemoglobin S, it is clear that the protective minor allele has reached polymorphic frequencies by conferring a survival advantage to carriers. Similar mechanisms can be invoked for most of the changes listed in table 2, but any such selection pressures on alleles that impact on non-infectious diseases would probably be far weaker.

Whatever the cause of the apparent excess of changes in exons associated with infectious diseases, arguably such coding changes are the associations one would most like to uncover, both to understand disease pathogenesis and also to provide further examples of selective pressures by infectious pathogens on human genetic diversity.

In conclusion, the new technology of exome sequencing offers an attractive approach to searching for numerous rare sequence changes that may associate with disease susceptibility. Moreover, initial data suggest that the impact (or effect sizes) of these uncommon coding changes may be greater than those of the higher frequency non-coding polymorphisms that are generally identified in GWAS.

## 9. Natural selection and infectious disease susceptibility genes

Case–control studies have provided the strongest evidence of ongoing selection by infectious pathogens on genetic polymorphism in humans. Less direct evidence of selection is provided by a variety of other statistical approaches to the analysis of genomic data, some of which are described in other chapters of this issue. A recent development is the use of a composite of multiple signals that can pinpoint variants likely to have undergone selection [74]. These regions and SNPs can then be prioritized for analysis in case–control studies of particular infectious diseases, illustrating the potential complementarity of these approaches.

However, some caveats in each approach should be noted. Some signals of selection extant in our genomes will have been generated by pathogens or strains of pathogens that have died out or been eradicated, preventing case–control studies of their effects, although *in vitro* studies or animal models might still allow some analysis and inferences. Conversely, the temporally varying natural history of epidemics and ever-varying genetic diversity of pathogens may tend to minimize signals of selection in our genomes, particularly when compared with the more continuous effects of other selective agents such as sunlight and climate. In this context, it is worth recalling that at least three types of selection have been demonstrated to be capable of maintaining genetic diversity in humans over long periods of evolutionary time, as is well illustrated by consideration of the maintenance of the extraordinary diversity of HLA genes in the human major histocompatibility complex. Although heterozygote advantage [75] and frequency-dependent selection [76,77] are the most frequently invoked modes of selection capable of maintaining polymorphic diversity, fluctuating selection pressures [76,78] (varying either spatially or temporally, or both) are also capable of maintaining polymorphism. Indeed, epidemiological records provide powerful evidence that fluctuating selection by lethal infectious pathogens is commonplace in recent human history and may always have been so, at least since the increase in population densities following the introduction of agriculture.

Such fluctuation in selection pressures will result not just from the transient occurrence of particular epidemics but from temporal and spatial changes in the antigenic composition of pathogens, as different strains are likely to interact differentially with particular host resistance genotypes. The impact on signatures of selection [79], as currently deduced from genomic sequence data, could be profound in that short periods of selection and then a reversal of the selection pressure would tend to minimize any detectable signals in the genome over time. As discussed earlier, several host genes affect resistance to multiple infectious pathogens and this too could tend to weaken signals if different pathogens interact differentially with various host genotypes. The overall effect of these complexities is that many statistical searches for genomic signatures of selection may underestimate the evolutionary impact of infectious pathogens, because of the complexity of selection involved, in contrast to simple directional selection pressures provided by some other factors such as climate or dietary change.

A major interest of the Discussion Meeting that led to this publication has been the use of human genetic variants as markers of migration routes and population differentiation in recent human evolution. Although some theoretical considerations would point to a preference for non-selected markers for this purpose, in practice, variants that have clearly been selected by infectious pathogens have proved very useful for tracing migration and population affinities. These include examples from the highly polymorphic HLA genes [80], and also specific haemoglobinopathies that were selected to high frequency by malaria but subsequently could be used to trace population migrations. For example, a specific alpha globin gene deletion in the southwest Pacific helped early genetic studies of the colonization of Oceania [81].

An overview of the infectious diseases that have received most attention by human geneticists and those that have produced the clearest successes provides a striking outlier. HIV, tuberculosis and malaria have probably had most investment and attention, in accordance with their global public health burden, but the star performer in terms of results must be leprosy. This was the first infectious disease shown to be HLA-associated [82], the most successful disease for mapping loci by linkage analysis [18–20] and, to date at least, the most successful example of a GWAS [32]. In the past, this may have reflected easier sample collection for leprosy but this is no longer the case. A more likely explanation may relate to the extreme lack of genetic polymorphism in the causative bacterium, *Mycobacterium leprae*, which has now been well documented by whole genome sequencing [83]. If this explanation is correct, then the implications may be profound. Many of the difficulties in mapping and identifying strong genetic factors for human infectious diseases may relate to the ‘pooling’ of genetically diverse strains of a single pathogen in studies of a single infectious disease. Hence, separating out cases according to strain or genotype of the pathogen may increase power to detect strain-specific host effects, and already there is some preliminary evidence that pathogen strain may influence infectious disease associations [84]. Now that full genome sequences of viral, bacterial and even some protozoan pathogens can be determined routinely, such an approach is becoming more feasible. Allowing for strain diversity in host genetic studies will undoubtedly increase complexity and the required sample sizes but may be particularly rewarding for studies of immunogenetic polymorphisms.

The study of the genetics of infectious disease susceptibility has undergone revolutionary change over the last decade, driven by spectacular advances in human genomics, and the rate of progress shows no sign of abating. Fascinating insights have been uncovered into the long battle between pathogens and their human host and the consequences for our genetic heritage. Although our power to understand and control major infectious pathogens is greater than ever, the true scale of the challenge posed by our need to maintain a long-term accommodation with the microbial world is only beginning to be appreciated.
